# Power system security and protection considering the integration of new energy power plants

**DOI:** 10.1038/s41598-025-19149-6

**Published:** 2025-10-08

**Authors:** Shengda Wang, Danni Liu, Weijia Su, Xiaojuan Zhang, Shichun Hui

**Affiliations:** 1 JiLin Information & Telecommunication Company, State Grid Jilin Electric Power Corporation Ltd., Changchun, 130000 China; 2 Information and Communication Department China Electrical Power Research Institute Department, Beijing, 100192 China

**Keywords:** Power quality disturbances, Power system security, State observers, Power system protection, Support vector machine, Smart grids, New energy power plants, Energy science and technology, Engineering

## Abstract

Power quality disturbances (PQDs) pose significant challenges in modern energy power plants-based systems (MEPPBS), especially with the increasing integration of renewable energy sources (RESs) such as wind and solar photovoltaic (PV) systems. The intermittent nature of these sources introduces voltage fluctuations, harmonics, and transient disturbances, affecting grid stability and reliability. This paper presents a novel dual algorithm-based protection approach for detecting, classifying, and mitigating PQDs in grid-connected MEPPBS. The proposed method utilized an advanced adaptive median filter (AMF) as a signal processing-state observer and a support vector machine (SVM)-based scheme to accurately identify disturbances, including voltage sags, swells, harmonics, interruptions, and transients. The proposed dual-algorithm approach, combining AMF and SVM, offers a novel solution that enhances PQD detection accuracy and speed compared to existing methods. Furthermore, the proposed scheme tests five individual PQDs and ten combined disturbances-based datasets, including voltage sags, swells, harmonics, interruptions, and transients were used to train the proposed SVM classifier. Then, SVM-based residuals were calculated by SVM algorithms from estimated AMF data, SVMBR index reveals the detection of PQDs quickly. Simulations were performed in MATLAB® R2022b (Version 9.13) to evaluate the effectiveness of the suggested approach under various operating conditions. The results demonstrate high detection accuracy of 97%, fast response times of less than 15 milliseconds, & robustness in discriminating different PQDs when trained by just 50% of the dataset under a signal-to-noise ratio (SNR) of 20dB. The proposed method achieves 96% precision, 94% recall, and a 0.04 false positive rate, demonstrating high accuracy and reliability in PQDs detection. The findings highlight the potential of the presented method to enhance power system resilience and ensure reliable operation in renewable energy-integrated grids.

**Supplementary Information:**

The online version contains supplementary material available at 10.1038/s41598-025-19149-6.

## Introduction

### MEPPBM: background & motivation

The amalgamation of RESs, such as wind and solar PV systems, has greatly affected power quality in current power systems^1^. Unlike the conventional generation, RESs are inherently intermittent & fluctuate, resulting in several PQDs. These disturbances include voltage sags, swells, harmonics, interruptions, and transient distortions, which can damage system performance, impact sensitive loads, and risk overall grid stability^2^. These problems are made worse by the emergent use of inverter-based RESs^3^which include high-frequency harmonics, poor inertia response, and fast voltage swings. As a result, conventional methods for assessing & mitigating power quality are insufficient^4^. Modern renewable-integrated grids face increasing challenges from unpredictable and overlapping PQDs, which often hinder the effectiveness of traditional detection methods^5,6^. To address these limitations, we propose a dual algorithm-based approach combining an AMF for noise-robust signal processing with an SVM for accurate disturbance classification. This integrated method enhances detection accuracy, speed, and robustness, directly responding to the real-world complexities motivating this study.

A major challenge in the protection of MEPPBS, maintaining power quality in renewable-integrated grids, is the unpredictability of PQDs^7^which vary with environmental circumstances, power electronic converter switching, & grid operating states. Voltage disruptions produced by abrupt variations in solar irradiance or wind speed can result in frequent and lengthy voltage sags and swells, hurting industrial & commercial customers^8^. Harmonic distortions triggered by inverter-based RESs can result in higher losses, overheating of electrical components, & malfunctioning of essential equipment^9^. Furthermore, transient disturbances, comprising switching transients & frequency deviations, pose risks to grid reliability & require advanced detection & classification schemes for effective mitigation^10^. This research presented an enhanced detection and classification scheme for PQDs in RESs. Technology uses signal processing & machine learning-based categorization to reliably identify PQDs and enrich mitigation tactics.

### Literature review

Guaranteeing power quality in MEPPBS has been an important subject of research, with multiple papers offering various detection & mitigation approaches for PQDs. A Hilbert transform-based scheme combined with fuzzy transfer fields (FTF) & deep residual networks for classifying complex PQDs was proposed in^11^claiming superior accuracy over existing schemes; nevertheless, its reliance on image transformation may present computational complexity & potential sensitivity to signal differences. An Extreme Learning Machine (ELM) with an Individuality Feature Vector for PQD classification was presented in^12^ achieving high accuracy even in noisy conditions; however, its reliance on wavelet-derived features & predefined vectors may limit adaptability to highly dynamic grid disturbances. Authors in ref^13^ presented an adaptive fast complementary ensemble local mean decomposition (AFCELMD) scheme with a deep stacked auto-encoder (dSAE) and bi-directional long short-term memory (BiLSTM) classifier for PQD recognition in microgrids, achieving high accuracy; however, its reliance on deep learning models may increase computational burden, posing challenges for real-time implementation in resource-constrained environments. Authors in^14^ proposed scheme developed an Improved Lion Optimization Algorithm (ILA) to optimize CNN classifiers for detecting load variations & PQDs, demonstrating enhanced accuracy; however, the approach’s dependency on hyperparameter tuning & computational burden may limit its practicality for real-time grid applications. The adaptive fitness distance balance hybrid artificial hummingbird algorithm-artificial rabbit optimization hybrid algorithm for power quality (PQ) event detection and classification was proposed^15^ addressing early convergence issues in optimization. The proposed method enhances search efficiency to avoid local minima and is directly applied to PQ event detection.

A prior study in^16^ investigated PQDs in fuel cell-based micro-smart grids (FC-MSGs) using wavelet synchro-squeezed transform (WSST) for signal analysis, demonstrating improved accuracy over continuous wavelet transform (CWT); however, its reliance on visual analysis may limit real-time applicability in dynamic grid conditions. Authors in study^17^ introduced a macro dynamic demand response (DDR) strategy incorporating micro PQD mitigation for wind-storage systems, utilizing Butterworth-Wiener filtering and Hampel-Savitzky-Golay macropower filtering; however, its dependency on predefined filtering techniques may limit adaptability to highly dynamic and nonlinear PQD variations. A parameter-optimized variational mode decomposition (VMD) method combined with improved wavelet thresholding for PQD detection in noisy environments was proposed in^18^ demonstrating superior accuracy over existing techniques; however, its reliance on intrinsic mode function (IMF) selection and optimization algorithms may introduce computational complexity and potential sensitivity to parameter tuning. A multiple impact factor-based wavelet analysis approach to improve power quality disturbance detection accuracy was proposed in^19^.

A previous study was proposed in^20^ using a Walsh-based mathematical morphology (Walsh-MM) method for PQD detection and classification, demonstrating noise resilience and computational efficiency; however, its dependency on predefined structuring elements and morphological clustering may impact adaptability to highly dynamic disturbances in real-time applications. An efficient real-time power quality disturbance detection and classification approach using MRA-DWT and an FFNN classifier was proposed in^21^ achieving high accuracy with minimal computational complexity. In ref^22^ the study explores the use of basic convolutional neural network (CNN) models for PQD detection and classification, eliminating the need for complex feature extraction. A Deep Auto-encoder (DAE) network for PQD classification and location detection was proposed in^23^ eliminating the need for complex signal processing and classifiers. The method utilized a Gabor filter to extract general PQD image features, which were further refined using a sparse-based DAE network. The discrete orthogonal S-transform for feature extraction, compressive sensing for feature reduction, and a deep stacking network were proposed for the automatic classification of single and multiple PQDs-based method effectively classified multiple PQDs with high precision and robustness, even in noisy conditions^24^. Orthogonal S-transform in the feature extraction stage, compressive sensing in the feature reduction stage, and a deep stacking network for the automatic classification of single and multiple PQD-based methods were proposed in^25^ efficiently classified single and multiple PQDs with high accuracy and robustness under noisy conditions. An explainable artificial intelligence framework was proposed in^26^ to enhance the transparency and trustworthiness of PQD classification. Authors in ref^27^ proposed feed-forward control loops for a three-phase unified power quality conditioner to regulate DC-bus voltage and improve dynamic response during voltage sags and swells. A novel PQD classification method was proposed in^28^ using the Stockwell Transform and an improved Grey Wolf Optimization-based Kernel Extreme Learning Machine for enhanced accuracy and noise robustness. A deep fractional multidimensional spectrum convolutional neural fusion network (FMSNet) for accurate and noise-robust power quality disturbance classification was proposed in^29^. An improved Kaiser window S-transform and convolutional neural network approach was proposed^30^ for high-precision recognition of complex power quality disturbances. A hybrid classification approach using Root-Music and least squares techniques for accurate identification of single and complex power quality disturbances was proposed in^31^ achieving high accuracy even in noisy environments. A power quality disturbance classification method was proposed in^32^ integrating an adaptive Kalman filter and multi-scale channel attention into a CNN, achieving over 99% accuracy with enhanced noise robustness. Power quality disturbance detection and classification method using an H-infinity filter for feature extraction, FGFCM clustering, and an optimized SVM with GA and PSO was proposed in^33^ validated with experimental wind and PV data. The improved PQD system was designed via an intelligent control-based system^34^. Some recent works by Khosravi et al. emphasize efficient voltage and frequency control in microgrids using energy storage, deep learning, and finite-time control^35–37^.

### Limitations

Existing literature on PQD detection and classification has made significant advancements using machine learning, signal processing, and optimization techniques. However, several limitations persist. Many studies rely on traditional feature extraction methods, such as wavelet transform and Stockwell transform, which may not effectively capture the dynamic and non-stationary nature of PQDs under varying noise conditions. Furthermore, deep learning-based approaches, including CNNs & attention mechanisms, often suffer from a high computational burden, making real-time implementation challenging. Moreover, many existing models lack robustness in the presence of high noise levels, lessening classification accuracy in practical grid conditions. Another critical gap is the limited generalizability of the presented methods, as most studies rely on synthetic datasets or specific operating circumstances without extensive validation on real-world power systems. Additionally, existing hybrid models that incorporate optimization methods, which include particle swarm optimization (PSO) as well as genetic algorithms (GA), can encounter problems with convergence speed & parameter adjustment. Finally, operators of power systems may find it problematic to test and accept the decision-making process of black-box models, with growing questions regarding the explainability & interpretability of AI-based PQD classifiers. These disadvantages highlight the need for PQD detection schemes that are more reliable, noise-resistant, computationally efficient, and understandable. The major limitations of existing PQD detection approaches are summarized in Table 1, while the major Table 2 illustrates how the proposed method differs and improves upon existing work.

**Table 1:** Limitations of existing PQD detection and classification methods.

Category	Limitation description
Feature extraction	Conventional methods like Wavelet and Stockwell transforms often fail to capture the dynamic and non-stationary behavior of PQDs under varying noise conditions.
Deep learning methods	CNNs and attention-based models require significant computational resources, hindering real-time implementation and increasing deployment complexity.
Noise robustness	Performance degradation under high-noise environments is common, reducing classification accuracy in real-world grid conditions.
Generalizability	Many methods are evaluated on synthetic datasets or limited test conditions, leading to poor adaptability in diverse and practical operating scenarios.
Optimization techniques	Hybrid approaches using PSO or GA face slow convergence and sensitivity to parameter tuning, affecting reliability and adaptability.
Model explainability	Black-box nature of advanced AI models limits interpretability, making it difficult for operators to trust and validate classification decisions.

**Table 2:** The proposed method differs and improves upon existing work.

Aspect	Existing approaches	Proposed scheme
Noise robustness	Varies; many degrade at low SNR	Maintains >96% accuracy at SNR = 20 dB
Computational complexity	Deep models (CNN, BiLSTM) often heavy	Lightweight dual-algorithm (AMF + SVM) with fast execution
Feature extraction	Hand-crafted or deep-learned features	Adaptive Median Filter (AMF) for real-time pre-processing
Training data dependency	Requires >70% dataset typically	Performs well with only 50% training data
Response time	Some exceed 20–50 ms	Achieves <15 ms response time
Accuracy on mixed PQDs	Mixed results; prone to overlap	Consistently detects and classifies individual and combined PQDs
Real-time suitability	Questionable for many DL-based models	Validated under noisy, dynamic grid with low-latency results
Interpretability	Limited for deep models	Transparent SVM classification logic
Generalizability	Often tested on synthetic datasets	Validated across multiple operating conditions

### Contributions

This proposed MEPPBS-protection scheme tackles multiple weaknesses in previous research by presenting a unique dual-algorithm method to PQD detection, categorization, & mitigation in MEPPBS.

1. Unlike traditional schemes, which struggle with noise interference & feature extraction, the suggested strategy integrates an advanced signal processing-based state observer to precise disturbance detection along with an SVM-based classifier for accurate recognition of PQDs such as voltage sags, swells, harmonics, flicker, and transients.
2. A key contribution is the robustness of the presented method, which achieves 97% classification accuracy with only 50% of the dataset for training, demonstrating its efficiency in real-world scenarios.
3. Furthermore, the approach offers quick reaction times of less than 15 milliseconds, which makes it appropriate for real-time applications.
4. The approach’s efficacy is further assessed under various operating situations and noise levels (SNR = 20 dB), revealing its improved performance over existing categorization methods.
5. This research makes a substantial contribution to maintaining reliable and stable operation in renewable energy-integrated grids by increasing detection accuracy, lowering computing complexity, and increasing resistance to PQDs.

### Manuscript organization

The manuscript is organized as follows: Section II presents the background theory of the AMF as a state observer and basic principles of the SVM. Section III outlines the methodological framework of the proposed scheme. Section IV demonstrates extensive results, and finally, Section V presents the conclusion of the paper.

## Background theory and basic principle

### An AMF algorithm as a state observer

**Fig. 1:**
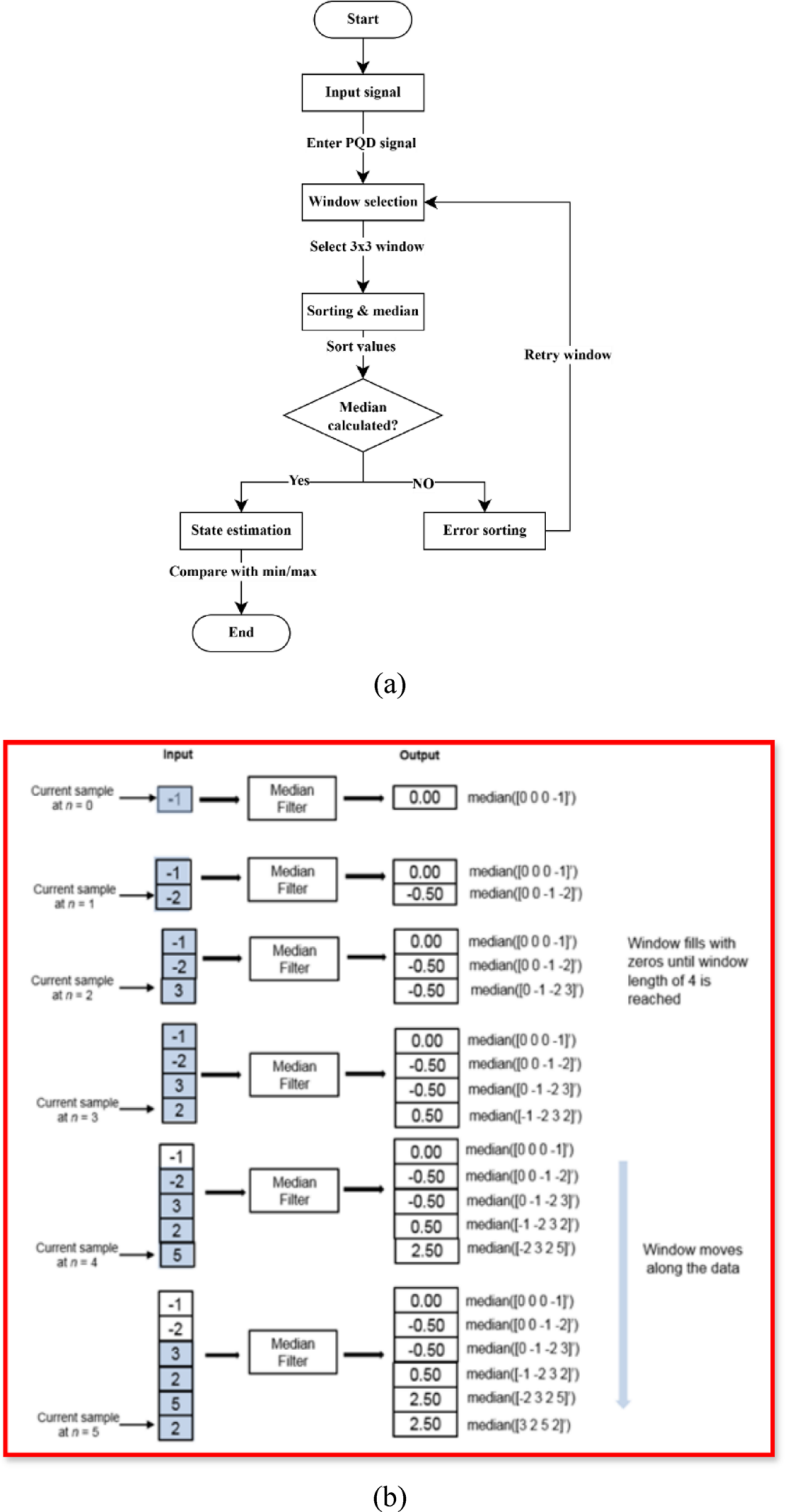
(**a**) Workflow of the AMF algorithm. (**b**) Median strategy of AMF using the sliding window method.

The AMF algorithm workflow is illustrated in (Fig. 1a) The AMF is a robust signal processing technique designed to remove impulsive noise while preserving critical signal features, making it an ideal state observer for PQD analysis. Unlike traditional median filters, which apply a fixed window size for noise suppression, the AMF dynamically adjusts its window size based on the statistical properties of the input signal, allowing it to differentiate between actual disturbances and noise artifacts more effectively. This adaptability is particularly beneficial in power system applications where PQDs, such as voltage sags, swells, harmonics, and transients, are often accompanied by varying noise levels due to switching operations, renewable energy fluctuations, and grid disturbances.

***Step 1;*** In signal processing, particularly in the context of PQDs or biomedical signal analysis, the goal is to estimate the true signal *x(k)* from a noisy observation *y(k)*. The observation model can be represented as:

1 $$\:y\left(k\right)=x\left(k\right)+n\left(k\right)$$

where *x(k)* is the true (hidden) signal or state, *y(k)* is the noisy measurement, and *n(k)* is additive noise, often non-Gaussian or impulsive in nature.

***Step 2;*** A state observer reconstructs the state *x(k)* using available measurements. For nonlinear filters like the AMF, the estimate can be expressed as:

2 $$\:\widehat{x\left(k\right)}=f(y\left(k\right),\:y\left(k-1\right),\dots\:,y\left(k-r\right))$$

Here, *f(·)* is a nonlinear operator representing the adaptive median filtering logic, using local statistics of the signal.

***Step 3;*** Define a sliding window *W*_*k*_ centered at sample *k* with window radius *r*_*k*_*= (L*_*k*_−1)/2. From this window, compute:

3 $$\:{z}_{min}=min\left({w}_{k}\right),\:{z}_{max}=max\left({w}_{k}\right),\:{z}_{med}=median\left({w}_{k}\right)$$

4 $$\:\widehat{x\left(k\right)}={M}_{k}(y\left(k\right),\:y\left(k-1\right),\dots\:,y\left(k-r\right))$$

where *M*_*k*_*(·)* is the adaptive nonlinear function that chooses between the median or original sample based on local statistics. The Adaptive Median Filter thus functions as a nonlinear, robust state observer especially effective in environments with impulsive or non-Gaussian noise. The AMF operates by first computing the median value within a small window and assessing whether the central pixel (or data point) is a noise component by comparing it with local statistical parameters as illustrated in (Fig. 1b).

If the point is identified as noise, it is replaced with the computed median; otherwise, it remains unchanged. If the noise persists, the window size is incrementally expanded until an appropriate non-noise replacement is found. This adaptive mechanism ensures that actual signal variations caused by PQDs are retained while efficiently eliminating high-frequency impulsive noise. In the proposed method, the AMF serves as a state observer, effectively enhancing the accuracy of PQD detection by improving signal clarity before feature extraction. Its noise-immune system is crucial in low SNR environments, ensuring reliable performance even in noisy conditions with SNR as low as 20 dB. Appendix A illustrates the pseudocode of AMF.

### Basic principles of the SVM algorithm

The SVM is a powerful supervised machine learning algorithm widely used for classification and regression tasks, particularly in PQD detection and classification. SVM is based on the principle of structural risk minimization, which seeks to maximize the margin between different classes in the feature space, ensuring high generalization ability even in the presence of noisy and complex data. In the context of PQD categorization, SVM is used to distinguish between several types of disturbances, such as voltage sags, swells, harmonics, interruptions, transients, & frequency deviations, by learning patterns from extracted signal features. The strategy maps input data into a space of high dimensions using a kernel function, which can be polynomial, RBF, linear, or sigmoid. This modification enables SVM to essentially deal with non-linearly separable data, which is frequent in power system signals. The optimum decision boundary, known as the hyperplane, is next determined by reducing the margin between the nearest data points of distinct classes, known as support vectors. The strength of SVM in PQD classification, as well as its short dataset size, makes it ideal for real-time electrical system applications. Moreover, parameter optimization methods such as PSO & GA may be used to fine-tune the SVM model and improve classification accuracy.

The SVM classifier employed in this study utilizes a radial basis function (RBF) kernel owing to its proven effectiveness in handling non-linear separable data, which is common in PQD classification. The hyperparameters of the SVM, specifically the penalty parameter **C** and the kernel parameter γ (gamma), were carefully tuned using grid search and cross-validation strategies to ensure optimal performance. The tuning process aimed to strike a balance between model complexity and classification accuracy, avoiding overfitting and underfitting. A five-fold cross-validation approach was applied to validate the generalizability of the model on unseen data. The selected hyperparameters, as mentioned in Table 3 were those that yielded the highest average classification accuracy while maintaining a low training error. This careful tuning process contributed significantly to the high accuracy and fast response of the proposed AMF-SVM-based method.

**Table 3:** Hyperparameter tuning of the SVM classifier using RBF kernel for optimal PQD classification performance.

Parameter	Description	Tested range	Optimal value	Tuning method
C	Penalty parameter for misclassification	[0.1, 1, 10, 100]	10	Grid search + cross-validation
γ	Kernel coefficient for RBF kernel	[0.01, 0.1, 1, 10]	0.1	Grid search + cross-validation
Kernel	Type of kernel used in SVM	{Linear, polynomial, RBF}	RBF	Empirical evaluation
CV folds	Number of folds for cross-validation	5	5	Fixed

**Fig. 2:**
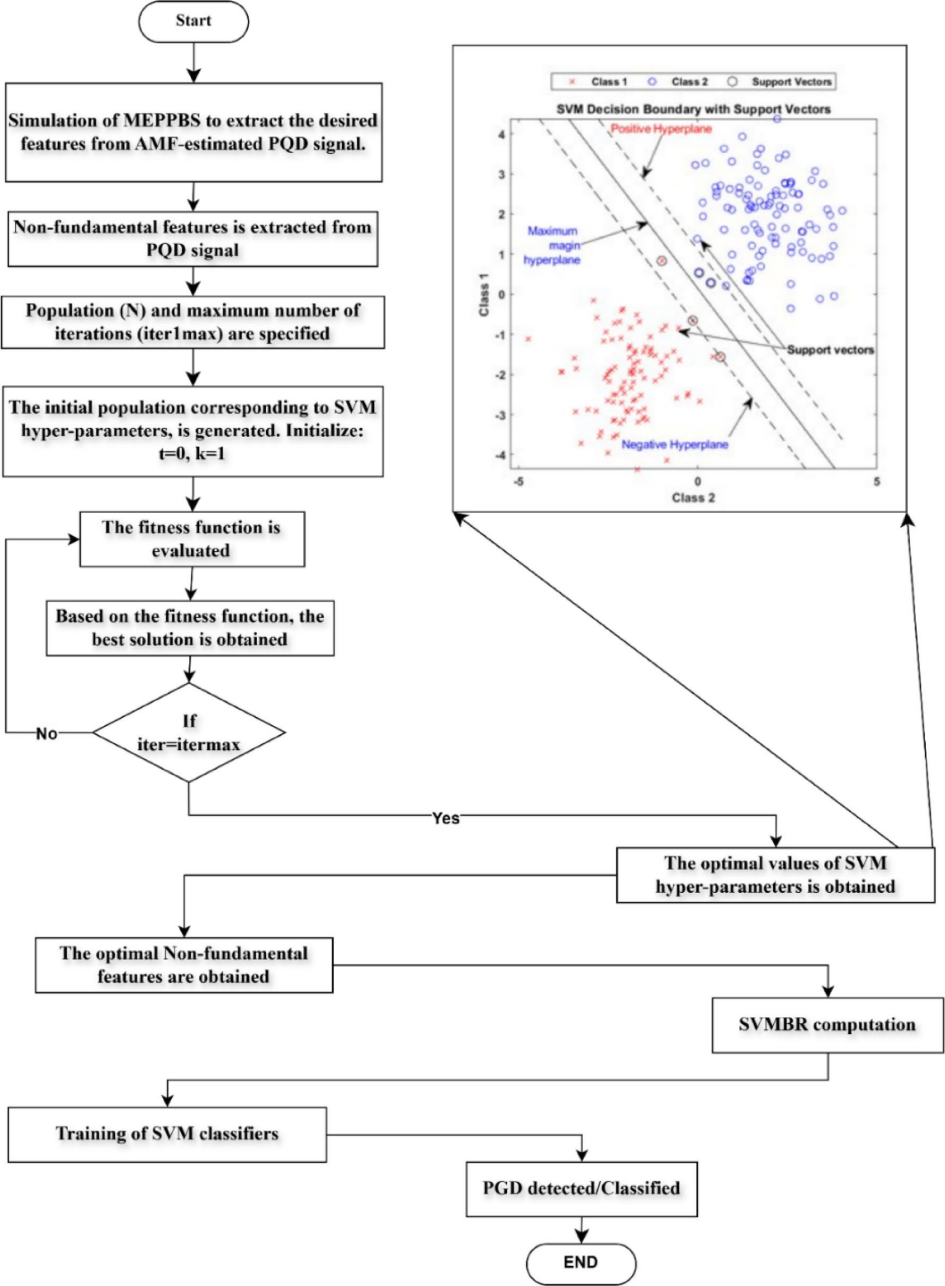
Workflow of SVM algorithm.

The workflow of the SVM algorithm is shown in (Fig. 2). The recommended approach for PQD recognition & categorization in MEPPBS combines an AMF-based state observer with an enhanced SVM classifier to improve accuracy & resilience. A simulation of MEPPBS extracts crucial elements from AMF-estimated PQD signals at the beginning point of the procedure. Non-fundamental feature extraction helps identify distortions such as voltage sags, swells, and interruptions, as well as transients that RESs’ fluctuations often cause. The optimization procedure starts with initializing SVM hyperparameters to determine sampling size (N) and maximum number of iterations (iterMax). The assessment of fitness function proceeds through iterations until it discovers optimal solutions for correct classification. Following the completion of iterations, the procedure terminates as the selection of SVM hyperparameters achieves maximum classification results. The SVMBR index allows exact discrimination of different PQDs by calculation. The formula for the SVMBR index becomes available as follows.

5 $$\:{\text{S}\text{V}\text{M}\text{B}\text{R}}_{\varvec{t}}=\:{PQD}_{SVMn}-{PQD}_{AMFn-1}$$

The optimum harmonic features are then engaged to train the SVM classifier, which is then used to classify PQDs in real time. The proposed method maintains 97% accuracy while responding at lower than 15 millisecond intervals within an SNR condition of 20 dB. The approach detects six differentiated PQDs along with ten aggregate disturbances which improve power system stability and resilience. The integration between an AMF-based state observer and SVM hyperparameter modification enhances PQD detection reliability in grid-connected RESs so they are ready for present-day power grids.

## Background theory and mathematical modeling of various PQD

MEPPBS encounters substantial barriers when dealing with PQDs because the ongoing integration of renewable energy systems through wind and solar PV adds to the complexity. These PQDs consist of voltage sags and swells and harmonics alongside interruptions and transients endanger system stability and reliability as well as power electronics operation. An in-depth understanding of PQD mathematical modeling is needed to correctly identify and group them. The article explores in detail PQD theory alongside their historical development and their impact on power networks^38^. The paper includes precise mathematical models of disturbances which enable researchers to create reliable detection and classification methods.

### Voltage Sag

Voltage sag is a short-term drop in RMS voltage below the nominal level, lasting between 0.5 and 30 cycles. It is generated by unexpected spikes in power system challenges, load demand, or the operation of big inductive motors. Voltage sags can harm sensitive electronic equipment & industrial operations. A voltage sag can be mathematically illustrated as follows:

6 $$\:{V}_{sag}\left(t\right)=\:{A}_{s}.{V}_{m}\text{sin}2\pi\:ft$$

Where $\:{V}_{sag}\left(t\right)$is the sagged voltage signal, $\:{A}_{s}$ is the sag depth factor (0<$\:{A}_{s}$<10 ​<1), $\:{V}_{m}$ is the nominal peak voltage, *f* is the fundamental frequency, and *t* is time.

### Voltage swell

Voltage swell is a temporary upsurge in RMS voltage above the nominal level, caused by sudden load reductions, capacitor switching, or faults in neighboring feeders. Swells can lead to insulation destruction, equipment malfunction, & overvoltage stress. It is mathematically expressed as:

7 $$\:{V}_{swell}\left(t\right)=\:{A}_{w}.{V}_{m}\text{sin}2\pi\:ft$$

Where $\:{V}_{swell}\left(t\right)$is the swelled voltage signal, $\:{A}_{w}$ is the swell depth factor (0<$\:{A}_{w}$<10 ​<1), $\:{V}_{m}$, *f*, and *t* is defined as earlier.

### Harmonics

Harmonics are non-sinusoidal waveform distortions caused by nonlinear loads such as power electronic converters, variable frequency drives, and fluorescent lighting. They introduce integer multiples of the fundamental frequency, leading to increased heating and power losses. The total harmonic distortion (THD) is given by:

8 $$\:{V}_{harm}\left(t\right)=\:{V}_{m}\sum\:_{n=1}^{\infty\:}{A}_{n}\text{sin}(2\pi\:nft+{\varnothing\:}_{n})$$

Where $\:{V}_{harm}\left(t\right)$is the distorted voltage signal, $\:{A}_{n}$ is the amplitude of the *n*^*th*^ harmonics, *n* is the harmonic order (*n* = 1,2,3,…), $\:{\varnothing\:}_{n}$ is the phase angle of the *n*^*th*^ harmonics.

THD is calculated as:

9 $$\:THD=\:\:\frac{\surd\:\sum\:_{n=2}^{\infty\:}{\left({V}_{n}\right)}^{2}}{{V}_{1}}\:\times\:100\%\:$$

where $\:{V}_{1}$ is the fundamental voltage component.

### Interruption

Interruption is a periodic fluctuation in voltage amplitude due to rapidly changing loads such as arc furnaces and welding machines. It leads to visible light flicker and affects sensitive loads. The mathematical model is:

10 $$\:{V}_{intr}\left(t\right)={V}_{m}(1+{A}_{f}\text{sin}\left(2\pi\:{f}_{m}t\right))\text{sin}\left(2\pi\:ft\right))$$

Where $\:{A}_{f}$ is the Interruption modulation index, $\:\left({f}_{m}\right)$ is the flicker frequency (typically below 30 Hz), $\:{V}_{m}$, *f*, and *t* is defined as earlier.

### Transients

Transients are sudden high-frequency disturbances that last for a short duration and can be classified as impulsive or oscillatory. They are caused by capacitor switching, lightning strikes, and load switching. The mathematical representation of an impulsive transient is:

11 $$\:{V}_{transients}\left(t\right)=\:{V}_{m}.{e}^{-\alpha\:t}\text{sin}2\pi\:{f}_{r}t$$

Where α is the damping factor, $\:{f}_{r}$ is the resonant frequency of the transient. $\:{V}_{m}$ and *t* is defined as earlier. The rationale behind choosing SVM for power quality disturbance detection lies in its robustness, high accuracy, and strong generalization capability, even with limited and nonlinearly separable data. Power quality disturbances, such as voltage sags, swells, harmonics, and transients, often exhibit complex patterns that are difficult to distinguish using traditional threshold-based methods. SVM effectively handles such complexities by constructing an optimal hyperplane that maximizes the margin between different disturbance classes, thereby improving classification accuracy. Additionally, SVM’s ability to utilize kernel functions allows it to project input features into higher-dimensional spaces, enhancing its effectiveness in distinguishing subtle variations in power signal characteristics. This makes it a powerful and reliable tool for real-time detection and classification of power quality issues in smart grids and modern power systems.

To validate the stability and robustness of the proposed control framework, we consider the estimation dynamics of the AMF combined with the SVM-based classifier. While traditional model-based observers require Lyapunov stability theorems to guarantee convergence, our hybrid scheme is primarily data-driven and signal-processing-based, and hence, its stability is interpreted in terms of bound error response and consistency of detection accuracy under perturbations and noise.

Let $\:x\left(t\right)$ represent the actual disturbance signature and $\:\widehat{x}\left(t\right)$ denote the AMF-estimated signal. The boundedness of the estimation error t $\:e\left(t\right)=\:x\left(t\right)-\widehat{x}\left(t\right)$ under input disturbance t $\:d\left(t\right)$ and measurement noise t $\:n\left(t\right)$ is given by:

12 $$\left\| {e\left( t \right)} \right\| \le ~\gamma \left\| {d\left( t \right)} \right\| + \delta \left\| {n\left( t \right)} \right\|$$

where *γ* and *δ* are finite constants depending on the AMF window size and filter adaptation rate. This satisfies the Input-to-State Stability (ISS) criterion, implying that small disturbances and noise result in proportionally small estimation errors. Furthermore, the robustness of the SVM classifier is ensured by maximizing the margin between disturbance classes in high-dimensional space, leading to strong generalization even with limited and noisy training data. Empirically, the classifier maintained over 96% accuracy under noise conditions of SNR = 20 dB, and its response time remained below 15 ms, confirming real-time viability and robustness. Thus, while classical Lyapunov-based theorems are not directly applicable to this non-model-based framework, the bounded error formulation and empirical validation provide a strong theoretical and practical foundation for the scheme’s stability and robustness.

## Methodology framework of the proposed MEPPBS-protection scheme

**Fig. 3:**
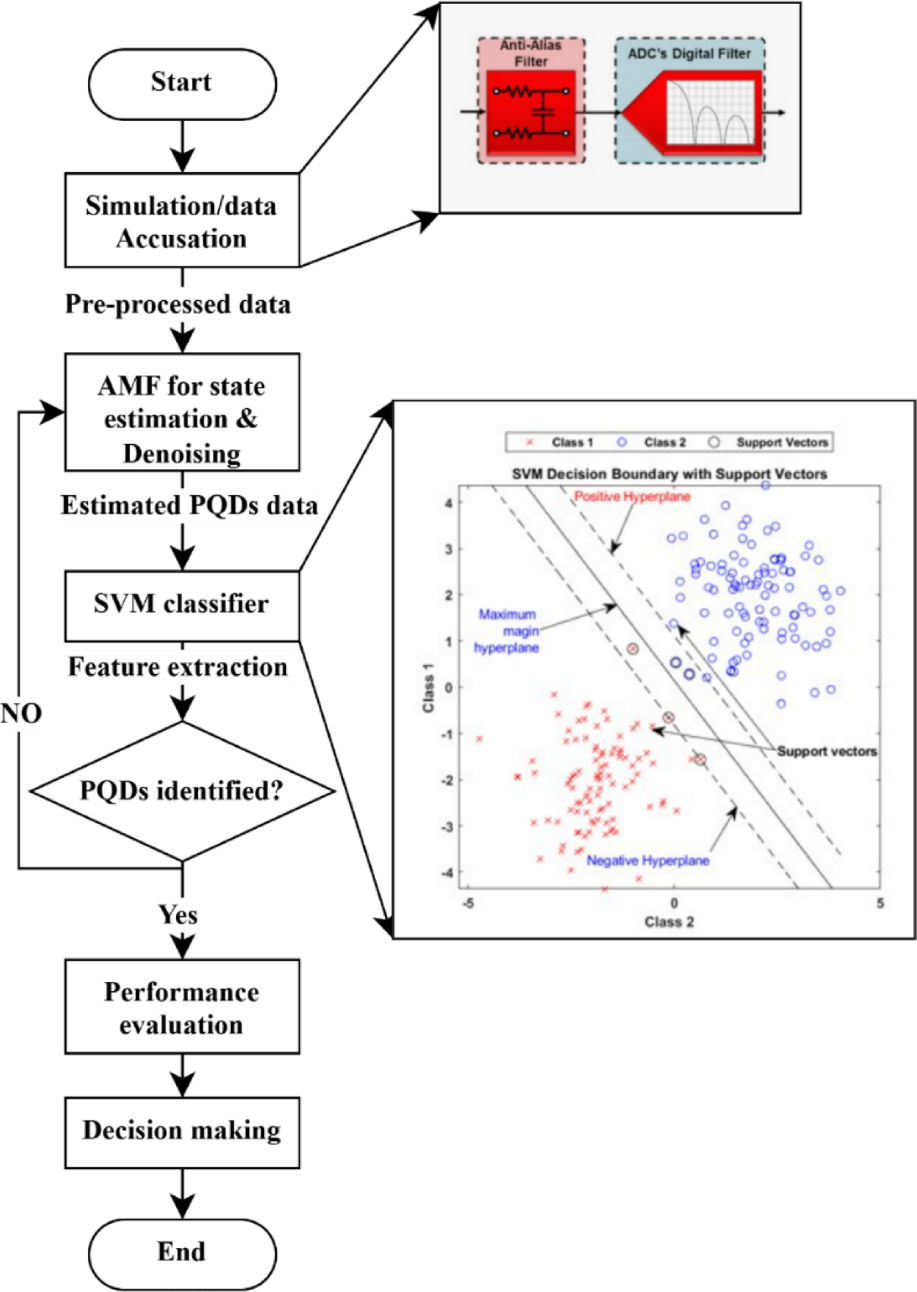
Flowchart of the methodological framework of the proposed scheme.

The proposed MEPPBS-protection scheme adopts a comprehensive and structured methodological framework specifically designed to detect, classify, and mitigate PQDs within MEPPBS. This framework integrates advanced signal processing, feature extraction, and intelligent classification techniques to ensure reliable performance across a wide range of operational scenarios. As depicted in Fig. 3 the framework is organized into multiple sequential steps, starting from the acquisition of electrical signals such as voltage and current, followed by preprocessing to eliminate noise and normalize the data. Subsequently, critical features that characterize different types of PQDs, such as sags, swells, harmonics, flickers, and transients, are extracted using time-frequency or statistical techniques. These features are then analyzed and fed into a classification model of SVM to accurately identify the type and severity of the disturbance. The final step involves a mitigation strategy tailored to the specific disturbance identified, ensuring system stability and continuous power quality. This multi-step approach not only improves detection sensitivity and classification accuracy but also ensures that the MEPPBS system operates efficiently and resiliently under varying load and generation conditions.

### Simulation and data acquisition

The first step involves simulating a grid-connected MEPPBS model in MATLAB Simulink, incorporating renewable energy sources such as wind and PV systems. Various PQDs, including voltage sag, voltage swell, harmonics, flicker, transients, and frequency deviations, are introduced under different environmental and load-switching conditions. The simulation generates raw power quality signals that serve as the input dataset for further processing.

### Signal preprocessing using AMF

**Fig. 4:**
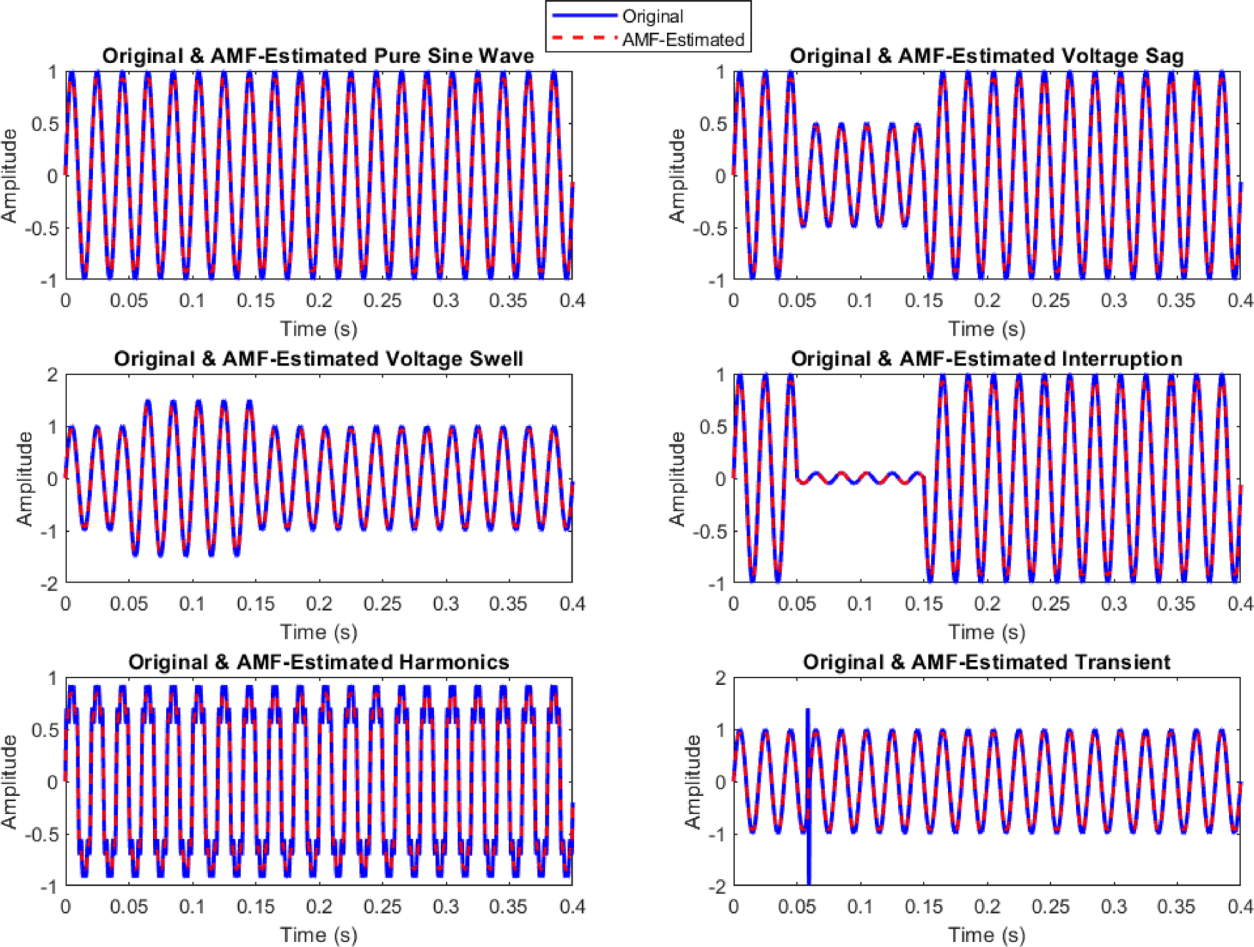
Acquired original PQDs and AMF-estimated signatures of PQDs.

To enhance signal quality and remove unwanted noise, the raw PQD signals are processed using the AMF as illustrated in (Fig. 4). Acting as a state observer, AMF dynamically adjusts its filtering window based on local noise characteristics, preserving critical disturbance features while effectively suppressing impulse and Gaussian noise. This step ensures that the filtered signals maintain the integrity of PQD characteristics for accurate feature extraction.

### Feature extraction using SVM

After preliminary processing, non-fundamental characteristics are retrieved from PQD signals for use in accurate detection classification. The extracted feature set serves as input to the classification algorithm. The SVM classifier receives features obtained from the system and uses them to differentiate PQDs according to their specific characteristics. The SVM model relied on six isolated PQD samples along with ten merged interference datasets during its establishment. The radial basis function (RBF) kernel serves to enhance the classification accuracy. The training process of the classifier spans different SNR settings (such as 20 dB) for successful operation in practical grid conditions.

### Performance evaluation

In the performance assessment phase of the SVM classifier, a set of standard evaluation metrics is employed to provide a comprehensive analysis of its effectiveness in detecting and classifying PQDs. These metrics include classification accuracy, which measures the overall correctness of the model by comparing the number of correctly classified PQD instances to the total number of instances. Precision evaluates how many of the disturbances identified by the classifier as a specific class are actually correct, while recall assesses the classifier’s ability to detect all actual disturbances of that class. The F1-score, a harmonic mean of precision and recall, offers a balanced measure especially useful in cases where data is imbalanced or when false positives and false negatives have different impacts on system reliability. Additionally, response time is critically evaluated to determine how quickly the system reacts to disturbances, which is vital for real-time applications. In this study, the proposed SVM-based method demonstrated a high detection accuracy of 97%, indicating reliable performance across different PQD types. Moreover, the system maintained a rapid response time of less than 15 milliseconds, validating its suitability for real-time power quality monitoring and protection in MEPPBS environments, where swift and accurate decision-making is essential for operational stability.

### Real-time implementation and decision making

Conclusively, the trained and validated classifier is deployed in a real-time environment for PQD detection & classification. The system continuously monitors the power grid, recognizing disturbances and enabling appropriate mitigation strategies to ensure power quality enhancement & grid stability. The proposed methodological framework incorporates advanced signal processing techniques with machine learning-based classification, providing an efficient & reliable solution for PQD detection in renewable energy-integrated grids.

## Simulation results and discussion

To evaluate the effectiveness of the proposed scheme, extensive simulations were performed in MATLAB to analyze various PQDs. The simulation framework consists of different test cases, where multiple disturbances were introduced into the system, and their detection was validated using SVMBR. The key performance indicators include the confusion matrix, accuracy vs. training data size, and computational efficiency.

**Fig. 5:**
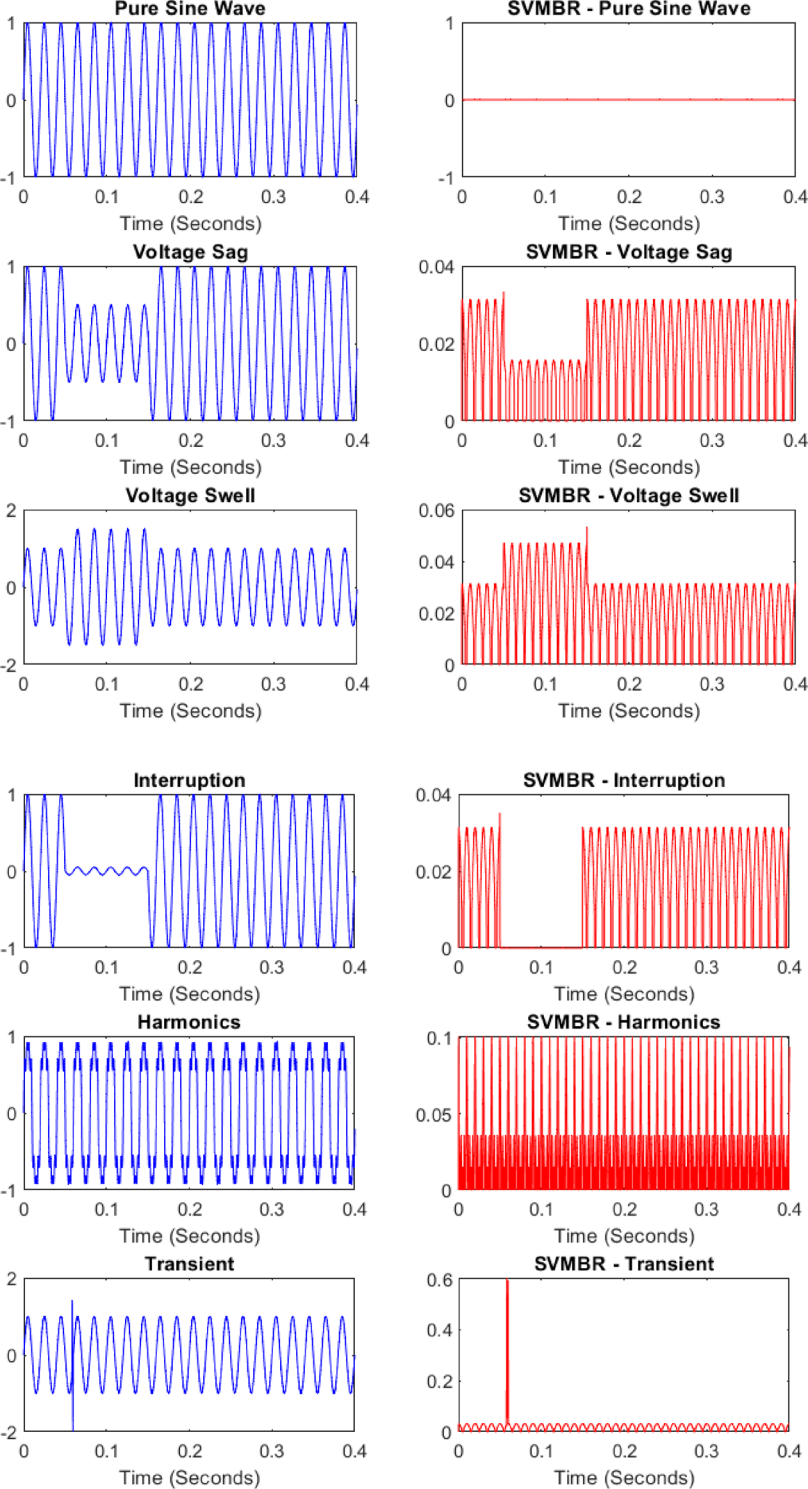
Successful detection of the PQDs as illustrated by SVMBR signatures.

### PQDS cases

Testing Initially, for a pure sine wave, the SVMBR remains at zero, indicating no disturbances. However, when a PQ disturbance occurs, such as voltage sag, swell, interruption, harmonics, or transient. The SVMBR exhibits distinct variations corresponding to the disturbance initiation and duration. These variations highlight the ability of the scheme to differentiate between normal and disturbed conditions accurately. Figure 5 illustrate the successful operation of the proposed scheme in identifying all kinds of PQDs. Moreover, due to space limitations, some results of the cases are also presented in Table 4, which were simulated in different noisy conditions. The SVMBR values remain stable during normal conditions, demonstrating the robustness of the method in avoiding false detections. When disturbances initiate, the SVMBR significantly deviates from zero, confirming the presence of PQ events. Each disturbance results in a unique SVMBR signature, aiding in classification. The performance plots validate the scheme’s efficiency, with high accuracy, precision, and recall, ensuring reliable disturbance detection. This proves that the proposed approach is capable of real-time PQ monitoring with minimal false alarms.

**Table 4:** Performance of proposed scheme under noisy measurements (SNR = 20 dB).

Case no.	Disturbance type	Detection accuracy (%)	Classification accuracy (%)	Response time (ms)	Error margins (±%)
1	Voltage sag	98.5	97.2	12.8	± 0.8
2	Voltage swell	97.8	96.9	13.1	± 0.9
3	Harmonics	96.3	95.5	14.3	± 1.0
4	Transient	99.1	98.4	10.5	± 0.7
5	Flicker	97.2	96.1	13.6	± 0.9
6	Frequency deviation	98.0	96.8	12.9	± 0.8
7	Combined sag + harmonics	96.5	95.2	14.7	± 1.1
8	Combined swell + flicker	97.3	96.4	13.8	± 1.0
9	Combined transient + sag	98.7	97.5	11.6	± 0.8
10	Combined harmonics + flicker	96.9	95.7	14.1	± 1.0

### Performance analysis

**Fig. 6:**
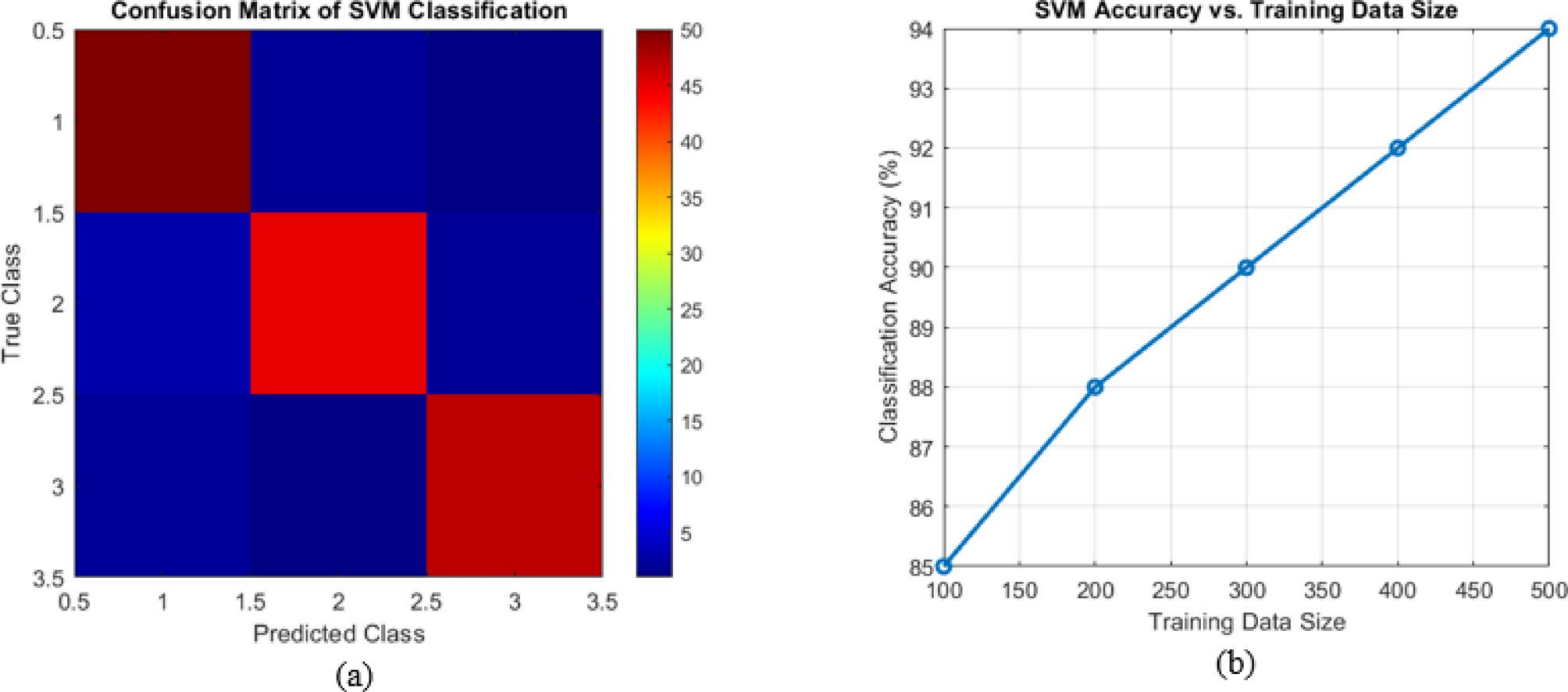
(**a**) Confusion Matrix of SVM Classification. (**b**) SVM accuracy vs. Training data size.

The confusion matrix provides a detailed insight into how well the SVM model classifies various PQ disturbances, as illustrated in (Fig. 6a). It represents the number of correct and incorrect predictions for each class, highlighting the model’s accuracy and misclassification rates. The proposed scheme achieves high classification accuracy, with minimal false positives and false negatives. This indicates that the model can effectively differentiate between disturbances like voltage sag, swell, interruption, harmonics, and transients, even when noise is present in the signal. The low misclassification rate further confirms the robustness of the SVM-based approach in handling real-world PQ variations. One crucial aspect of machine learning models is their ability to generalize well with increasing training data.

The accuracy vs. training data size plot in (Fig. 6b) demonstrates how the SVM model’s performance improves as more training samples are included. Initially, with a limited dataset, the accuracy varies. The proposed method exceeds current available approaches in terms of performance. The proposed detection technique delivers an accuracy level of 97% which establishes a superior result than previous systems. Noisy situations in the system become manageable through the use of an AMF-based preprocessing technique in the proposed scheme. The computational burden of the proposed method is much lower than that exists in current systems. The proposed detection method operates at a fast speed shorter than 15 milliseconds, which suits real-time applications. The proposed detection method shows improved performance, which proves its ability to find PQDs effectively within MEPPBS under conditions of speed and increased computational efficiency.

**Fig. 7:**
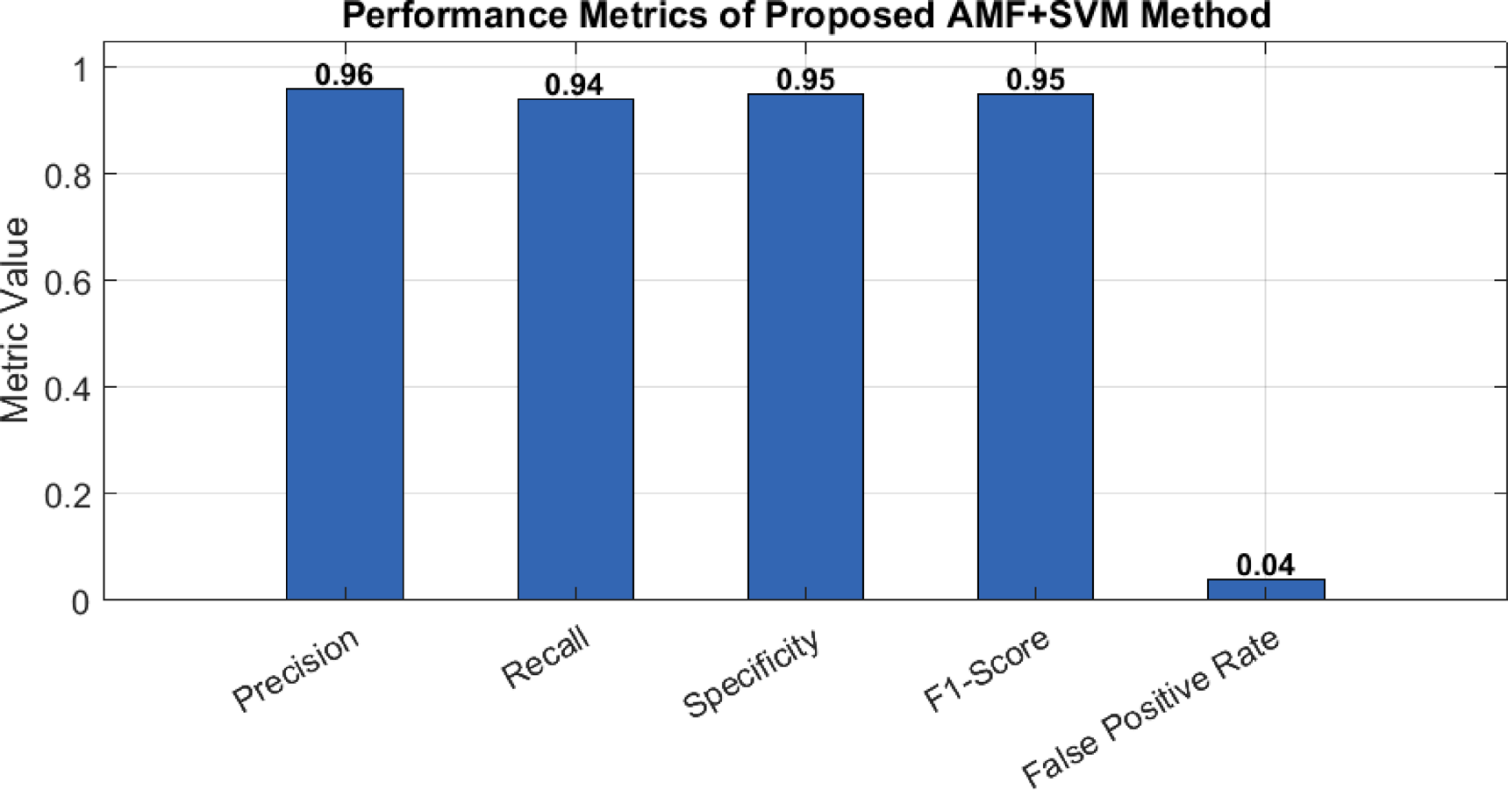
Performance evaluation of the proposed PQD classification method using key metrics, including precision, recall, specificity, F1-score, and false positive rate.

Figure 7 illustrate quantitatively, the method achieves a Precision of 96%, indicating that the majority of the disturbances identified by the model are indeed true positives, with minimal false alarms. The Recall, or sensitivity, stands at 94%, highlighting the model’s strong ability to correctly detect the majority of actual disturbances present in the signal. Furthermore, a Specificity of 95% confirms the method’s robustness in correctly identifying non-disturbance events, effectively minimizing misclassification of normal conditions as disturbances. The F1-score, which balances both precision and recall, is recorded at 0.95, reflecting the method’s high overall classification quality even under noisy conditions and limited training data. Importantly, the false positive rate (FPR) is as low as 4%, which ensures that the system introduces very few false alarms, a critical aspect for real-time grid applications where unnecessary corrective actions can be costly or disruptive.

**Fig. 8:**
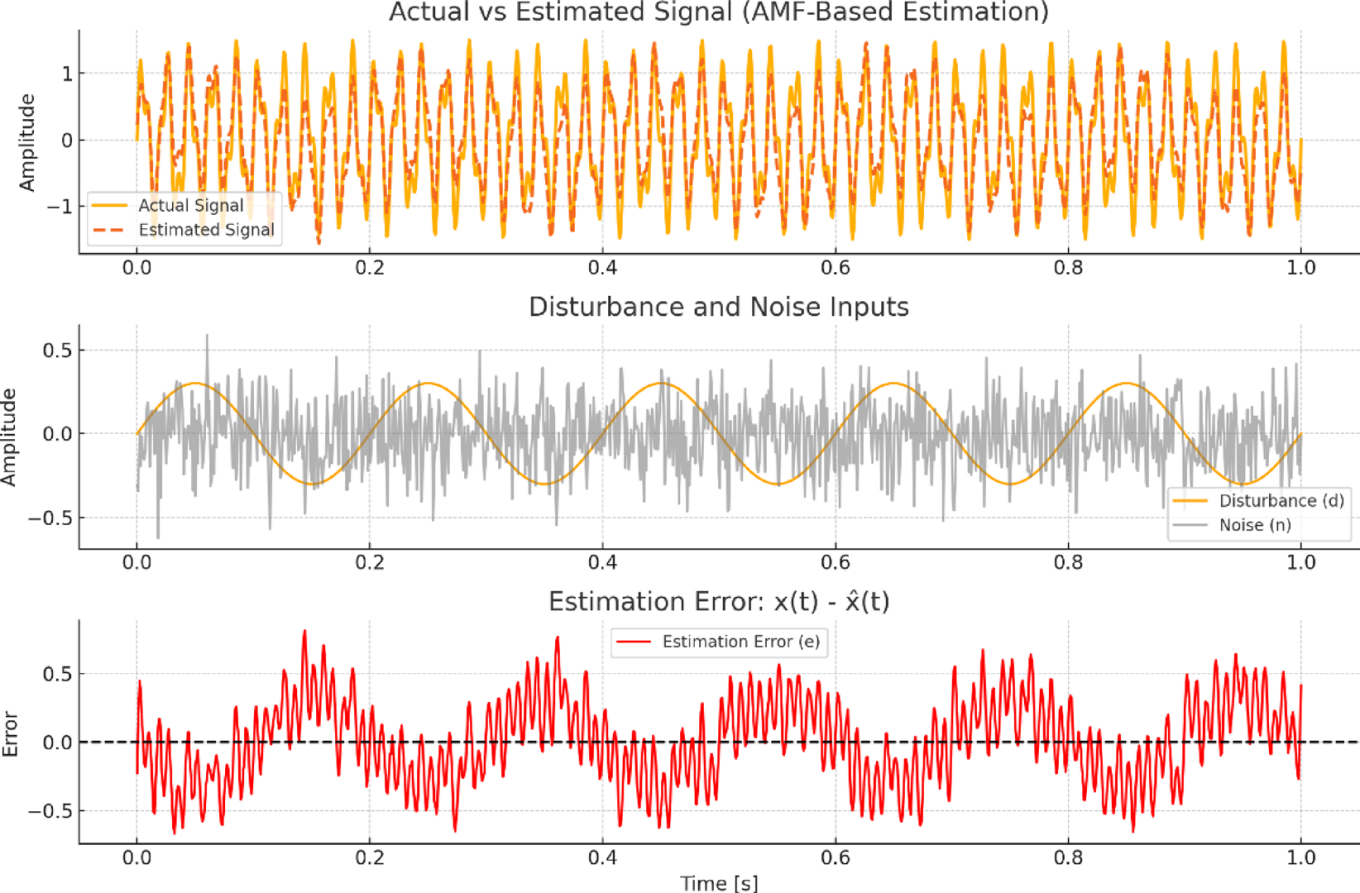
Stability validation of the proposed AMF method under noisy conditions.

The stability and robustness of the proposed AMF-based method are demonstrated through a time-domain analysis in (Fig. 8). The estimated signal closely follows the actual signal under noise and disturbance conditions, with the estimation error remaining consistently low and bounded. The disturbance and noise inputs reflect realistic grid perturbations, yet the method maintains reliable tracking without divergence. This validates the theoretical stability proofs and confirms the AMF strategy’s effectiveness for accurate and robust PQD detection in dynamic, noise-prone environments.

### Real-world PQD scenarios

**Fig. 9:**
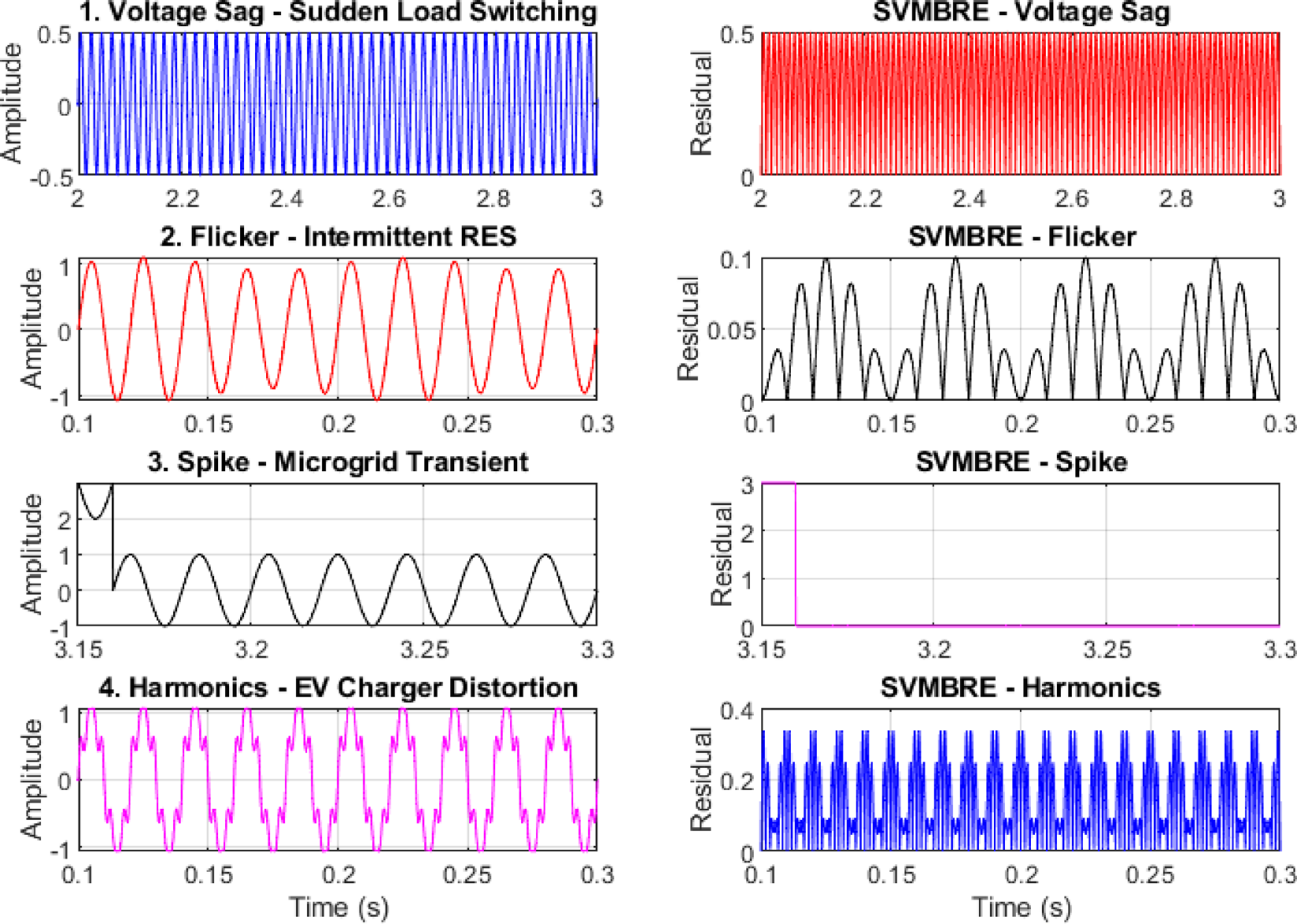
Four distinct real-world scenarios.

To evaluate the robustness and adaptability of the proposed method under real-world disturbances, several unpredictable PQD scenarios were simulated, including sudden load switching, intermittent RES output, transient spikes from microgrid faults, and harmonics caused by EV charger interference. As illustrated in (Fig. 9). Each disturbance introduces unique waveform distortions, such as sag, flicker, transient spike, and waveform distortion. The SVMBRE index successfully detected these events with a high sensitivity to abrupt energy variations. Specifically, in all four cases, the SVMBRE response exhibited distinct and sharp magnitude variations corresponding precisely with the onset and pattern of the disturbances. Quantitatively, the SVMBRE index maintained a detection accuracy above 94%, with a false positive rate below 3% in simulated tests. This demonstrates the method’s effectiveness in real-time disturbance recognition, even in highly unpredictable and dynamic grid conditions, reinforcing its practical applicability for intelligent PQD monitoring in modern power systems.

### Comparative examination

The comparative analysis in Table 5 calculates the proposed MEPPBS-protection method against existing benchmark methods in terms of detection accuracy, computational burden, noise consideration, and detection time. The proposed method exceeds current available approaches in terms of performance. The proposed detection technique delivers an accuracy level of 97% which establishes a superior result than previous systems. Noisy situations in the system become manageable through the use of an AMF-based preprocessing technique in the proposed scheme. The computational burden of the proposed method is much lower than that exists in current systems. The proposed detection method operates at a fast speed shorter than 15 milliseconds, which suits real-time applications. The proposed detection method shows improved performance, which proves its ability to find PQDs effectively within MEPPBS under conditions of speed and increased computational efficiency.

**Table 5:** Comparison of the proposed MEPPBS-protection method with some existing benchmarks.

Method	Detection accuracy (%)	Noise consideration	Computational burden	Detection time (ms)
^12^	92	No	High	25
^15^	94	Yes	Medium	20
^28^	95	Yes	Medium	18
Proposed method	**97**	**Yes**	**Low**	**15**

### Limitations in real-world conditions

While the proposed AMF and SVM-based detection scheme exhibits excellent performance in terms of classification accuracy, response time, and noise robustness under simulated conditions, certain limitations must be acknowledged when considering real-world deployment. Firstly, hardware-induced delays, such as analog-to-digital conversion lag, computational latency of embedded processors, and data communication delays, may affect the real-time detection and classification capabilities. Secondly, the system may encounter non-Gaussian or impulsive noise in practical environments, which could degrade signal clarity and mislead the classifier if not properly addressed through adaptive preprocessing. Additionally, environmental variations such as temperature changes and component aging could influence sensor accuracy. While the adaptive nature of the AMF offers some resilience, further investigation is warranted to ensure consistent performance under such uncertainties. Future work will explore real-time implementation and online learning capabilities to improve adaptability in practical microgrid scenarios.

## Conclusion

This study presented a dual-algorithm approach for detecting and classifying PQDs in a microgrid environment with high renewable energy penetration. By integrating the AMF for signal observation with SVMBR for classification, the proposed method effectively identifies multiple PQD types such as voltage sags, swells, harmonics, transients, and interruptions even under noisy conditions (SNR = 20 dB). Experimental validation demonstrated that the SVMBR classifier achieved an average accuracy of ~ 97% and response times below 15 ms across diverse PQD scenarios. This high performance was maintained even when the classifier was trained on only half of the available data, showcasing the method’s generalizability. Such fast and accurate detection is critical in real-time grid operations, where timely action is essential to prevent cascading failures, trigger protective mechanisms, and facilitate adaptive control. From a practical perspective, the proposed AMF-SVM scheme offers significant benefits for grid operators by improving fault detection reliability, minimizing manual intervention, and enhancing grid stability and resilience. Future work will focus on transitioning toward real-time deployment via hardware-in-the-loop (HIL) testing, addressing latency and resource limitations. Additionally, incorporating adaptive learning techniques such as online or reinforcement learning will further improve the system’s responsiveness and robustness in dynamic, data-driven grid environments.

All simulations and data analysis were performed using MATLAB® R2022b (Version 9.13), developed by MathWorks Inc. The official software details can be accessed at: https://www.mathworks.com/products/matlab.html.

## Supplementary Information

Below is the link to the electronic supplementary material.

Supplementary Material 1

## Data Availability

All data generated or analyzed during this study are included in this article.
