# Supplementary material for: Power system security and protection considering the integration of new energy power plants
Source: Sci Rep. 2025 Oct 8;15:35170. doi: 10.1038/s41598-025-19149-6 (PMC12508190; doi:10.1038/s41598-025-19149-6)
Supplement: Supplementary file 1 — Supplementary Material 1 [file 41598_2025_19149_MOESM1_ESM.docx]

**All simulations and data analysis were performed using MATLAB® R2022b (Version 9.13), developed by MathWorks Inc.. The official software details can be accessed at:** [**https://www.mathworks.com/products/matlab.html**](https://www.mathworks.com/products/matlab.html)**.**

**Appendix A (pseudocode code of AMF)**

classdef (StrictDefaults)MedianFilter < dsp.MedianFilter

methods

function obj = MedianFilter(varargin)

obj@dsp.MedianFilter(varargin{:});

end

end

methods (Access = protected)

%% Block Icon

function icon = getIconImpl(~)

icon = getString(message('dsp:StatisticsBlockDialog:MedianFilterIcon'));

end

%%Propagators

function cplx = isOutputComplexImpl(obj)

% Complexity propagator

cplx = propagatedInputComplexity(obj, 1);

end

function sz = getOutputSizeImpl(obj)

% Output size propagator

sz = propagatedInputSize(obj, 1);

end

function dt = getOutputDataTypeImpl(obj)

% Output datatype propagator

dt = propagatedInputDataType(obj, 1);

end

function isvar = isOutputFixedSizeImpl(obj)

isvar = propagatedInputFixedSize(obj,1);

end

function name = getInputNamesImpl(~)

name = '';

end

function name = getOutputNamesImpl(~)

name = '';

end

function val = supportsMultipleInstanceImpl(~)

% Support in for-each subsystem

val = true;

end

end

methods(Static, Access=protected)

%% Block header - widget grouping

function header = getHeaderImpl

% MATLAB System block header

header = matlab.system.display.Header(...

'dsp.simulink.MedianFilter', ...

ShowSourceLink=true, ...

Title='dsp:StatisticsBlockDialog:MedianFilterTitle',...

Text='dsp:StatisticsBlockDialog:MedianFilterHeader');

end

function mainS = getPropertyGroupsImpl

%Get default parameters group for this System object

propertyList = {

matlab.system.display.internal.Property('WindowLength',Description='dsp:StatisticsBlockDialog:WindowLength_MP'), ...

};

mainS = matlab.system.display.Section(...

Title = 'dsp:system:Shared:Parameters', ...

PropertyList = propertyList);

end

end

end
